# Comparative Analysis of Nuclear (*Ef1α*) and Mitochondrial (mt‐*CO1* and mt‐*Nad5*) Markers for Molecular Characterization of Sheep Isolates of Echinococcus granulosus sensu lato

**DOI:** 10.1002/vms3.70313

**Published:** 2025-03-20

**Authors:** Seyma Gunyakti Kilinc, Harun Kaya Kesik, Figen Celik, Sami Simsek

**Affiliations:** ^1^ Department of Parasitology Faculty of Veterinary Medicine Bingol University Bingol Turkey; ^2^ Department of Parasitology Faculty of Veterinary Medicine Firat University Elazig Turkey

**Keywords:** *ef1α*, *E. granulosus* s.s. (G1/G3), haplotype, sheep, mt‐*CO1*, mt‐*Nad5*

## Abstract

**Background:**

*Echinococcus granulosus* is a zoonotic disease that is widespread worldwide.

**Objective:**

This study aimed to determine the genetic diversity of *E. granulosus* isolates in sheep. Partial mitochondrial and nuclear gene sequences were used to determine intraspecific variation.

**Methods:**

For this purpose, 41 sheep hydatid cyst isolates were collected from slaughterhouses in Elazig and Bingol provinces of Türkiye. Following genomic DNA isolation from the germinal membrane of the cysts, PCR amplification and subsequent DNA sequence analysis were performed using primers that amplify mitochondrial *CO1*, *Nad5*, and nuclear *ef1α* gene regions.

**Results:**

DNA sequence analyses of mt‐*CO1* (1603 bp) and mt‐*Nad5* (625 bp) gene regions showed that 39 out of 41 isolates were identified as G1 and two isolates as G3. When the phylogenies formed by the sequences belonging to different gene regions were evaluated; in the phylogenetic tree created with the mt‐*CO1* and *Nad5* gene dataset, G1 and G3 isolates were in separate clades with the reference sequences, while in the phylogenetic tree created with the nuclear gene region *ef1α* dataset, the isolate belonging to the G3 genotype (ef1a.01) formed a sister clade with the G1 genotypes and the reference sequence. According to the haplotype network results obtained, 14 haplotypes, 15 haplotypes, and two haplotypes were determined for mt‐*CO1*, mt‐*Nad5*, and *ef1α* gene regions, respectively. Haplotype analysis of mt‐*CO1* and mt‐*Nad5* gene regions revealed high haplotype and low nucleotide diversity. Low nucleotide diversity was detected, and two haplotypes were determined as a result of haplotype analysis *ef1α* (1343 bp) gene.

## Graphical abstract

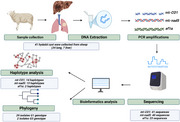

## 1 Introduction

*Echinococcus granulosus* is one of the most critical zoonotic parasites responsible for cystic echinococcosis (CE) in humans and animals around the world, being common in rural populations of humans and causing considerable economic loss, especially in undeveloped countries (Thompson 2017). Adult parasites reside in the small intestines of dogs, jackals, wolves, and other canids, whereas the larval hydatid cysts occur in sheep, goats, cattle, pigs, and numerous other domestic and wild mammals, as well as in different organs and tissues, particularly in the liver and lungs of humans (Simsek et al. 2006). In addition to its zoonotic importance, CE was included in the list of 117 diseases of great importance for animal health by the World Health Organization (WHO 2020).

Looking at the current classification of the genus *Echinococcus*, it is seen that there are five accepted species: *E. granulosus* sensu lato, *E. multilocularis*, *E. oligarthrus*, *E. vogeli*, and *E. shiquicus*. *E. granulosus* s.l. complex includes *E. granulosus* s.s. (G1/G3), *E. equinus* (G4), *E. ortleppi* (G5), *E. canadensis* (G6/7 and G8‐G10), and *E. felidis* species (Vuitton et al. 2020). Unlike previous studies, where sequences of mitochondrial cytochrome c oxidase 1 (mt‐*CO1*) and nicotinamid adenin dehydrogenase subunit 1 (nad1) gene regions have proven insufficient for discerning G1 from G3 genotypes, a much shorter region (759 bp) of mitochondrial NADH dehydrogenase subunit 5 (*Nad5*) gene has been determined to be sufficient, containing six informative positions, allowing for clear distinction of G1 and G3 genotypes (Kinkar et al. 2018a).

1 **Summary**

- A total of 41 hydatid cysts were collected from sheep. The PCR products were sequenced for mt‐*CO1*, *Nad5*, and *ef1α* genes.
- Thirty‐nine of 41 isolates were identified as *E. granulosus* G1 and 2 as G3 genotype.
- Fourteen haplotypes, 15 haplotypes, and two haplotypes were determined for mt‐*CO1*, mt‐*Nad5*, and *ef1α* gene regions, respectively.

Mitochondrial DNA (mt‐DNA) is frequently emphasized as a valuable source of genetic markers due to its haploid nature, rapid evolution, homoplasmy, and lack of recombination. However, mt‐DNA does not reflect the evolutionary history of the entire species since it usually originates from the maternal lineage (Giles et al. 1980; Avise et al. 1987). To classify species more efficiently, various loci from the nuclear genome can be analyzed (Saarma et al. 2009). Specifically, limited studies have been conducted to understand the phylogeny of *E. granulosus* s.l. using multiple nuclear loci, often yielding conflicting results (Saarma et al. 2009; Knapp et al. 2011; Laurimäe et al. 2018). Saarma et al. (2009) conducted a study using five different nuclear genes (ezrin‐radixin moesin‐like protein, elongation factor 1 alpha, transforming growth factor beta receptor kinase, thioredoxin peroxidase, and calreticulin) to perform a phylogenetic analysis of the genus *Echinococcus*. These analyses were found to be consistent with existing species classifications but revealed a significantly different phylogeny. As the nuclear data did not show a distinction between the G1 and G3 genotype clusters, it was concluded that G1 and G3 can be considered a single species (*E. granulosus* s.s.) and are separate genotypes only in the context of mitochondrial data (Laurimäe 2018). In this context, it is important to use both mitochondrial and nuclear gene regions to evaluate genetic diversity within the genus *Echinococcus* for the phylogenetic positioning of new *Echinococcus* spp. isolates (Saarma et al. 2009).

This study aimed to characterize the genetic diversity of *E. granulosus* s.l. isolates from sheep using mitochondrial and nuclear markers and to evaluate their contribution to the phylogeny of *Echinococcus*.

## 2 Materials and Methods

### 2.1 Collection of Hydatid Cyst Materials

Hydatid cyst materials (germinal membranes) were collected post‐slaughter from adult sheep over 1 year old at abattoirs in Bingol and Elazig provinces, eastern Türkiye. The liver, lungs, and other internal organs were examined for hydatid cysts. In animals afflicted with numerous cysts, non‐calcified cysts were chosen. Larger cysts were concurrently chosen because of the increased quantity of germinal membranes. Fluid from cysts was drained, and the germinal membrane was extracted, preserved in 70% ethanol, and transported to the laboratory. Each animal contributed a single cyst sample.

### 2.2 Genomic DNA Isolation, PCR, and Sequence Analyses

From the cyst samples preserved in 70% ethanol in the laboratory, a small piece of the germinal membrane was placed on a clean slide, cut into smaller pieces, and washed four to five times with 1X PBS (pH 7.4) to remove ethanol. After the final wash, gDNA isolation was carried out as per the recommendations of the PureLink Genomic DNA Mini Kit (Invitrogen, Thermo Fisher Scientific, Missouri, TX, USA). PCR was subsequently performed for each isolate using specific primers (Table 1) targeting the mitochondrial cytochrome oxidase c subunit 1 (*CO1*), mitochondrial NADH dehydrogenase subunit 5 (*Nad5*), and elongation factor 1 alpha (*ef1α*) gene regions of *E. granulosus* s.l. In a total volume of 50 µL, we added 5 µL 10X PCR buffer, 5 µL 25 mM MgCl_2_, 250 µM each of deoxinucleotides, 1.25 U Taq DNA polymerase, 20 pmol each of primer pairs, and 200 ng of template gDNA to the PCR mixture. PCR conditions for the mt‐*CO1* gene region included pre‐denaturation at 96°C for 6 min, followed by 35 cycles of denaturation at 94°C for 1 min, annealing at 54°C for 1 min, elongation at 72°C for 2 min, and a final elongation at 72°C for 10 min (Hüttner et al. 2008). PCR conditions for the *Nad5* gene region were optimized according to Kinkar et al. (2018a). This included pre‐denaturation at 95°C for 5 min, followed by 35 cycles of 45 s denaturation at 95°C, 45 s annealing at 55°C, 1 min elongation at 68°C, and a final elongation at 68°C for 5 min. PCR conditions for the *ef1α* gene fragment were performed as per the touchdown protocol described by Saarma et al. (2009). All PCR products were electrophoresed in agarose gel and visualized in a UV transilluminator to check for the presence of bands. A 100 bp marker (GeneDireX H3 RTU Cat. No. DM003‐R500) was used to determine the molecular weight of the bands.

**TABLE 1:** Specific primers amplifying mt‐*CO1*, mt‐*Nad5*, and *ef1α* gene regions of *E. granulosus*.

Gene region	Primer name	Primer sequence	Length of target size (bp)
mt‐*CO1*	F/CO1	5’‐TTACTGCTAATAATTTTGTGTCAT‐3’	1603 bp (Hüttner et al. 2008)
	R/CO1	5’‐GCATGATGCAAAAGGCAAATAAAC‐3’	
mt‐*Nad5*	EGnd5F1	5′‐GTTGTTGAAGTTGATTGTTTTGTTTG‐3′	759 bp (Kinkar et al. 2018)
	EGnd5R1	5′‐GGAACACCGGACAAACCAAGAA‐3′	
*ef1α*	EfF	5′‐ TCATTGTTATCGGTCACGTC ‐3′	1343 bp (Saarma et al. 2009)
	EfR	5′‐ CTTCTGGGCAGATTTTGTG ‐3′	

### 2.3 Phylogeny and Haplotype Network Analysis

After conducting DNA sequence analysis on the samples that yielded bands of the expected size from the PCR results, the alignment table and subsequent phylogenetic tree were generated using the MEGA X program (Kumar et al. 2018). The BLAST Search method at NCBI was employed to compare the results with published sequences. All sequence data were then uploaded to DnaSP 6 (Rozas et al. 2017). Population diversity indices—including haplotype number (h), haplotype diversity (Hd), and nucleotide diversity (π)—as well as neutrality indices such as Tajima's *D* (Tajima 1989), Fu's statistics (Fu 1997), and Fu and Li's *D* and F tests (FLD, FLF) (Fu et al. 1993) were calculated through DnaSP 6. Haplotype networks were created using the PopART‐1.7 software (Leigh et al. 2015).

## 3 Results

During the study, 41 sheep hydatid cyst materials were gathered from slaughterhouses in Bingol and Elazig provinces. Germinal membranes of 30 lung cysts and four liver cysts were collected from Elazig, while four lung cysts and three liver cysts were obtained from Bingol.

### 3.1 mt‐*CO1* Sequences and Haplotype Findings

**FIGURE 1:**
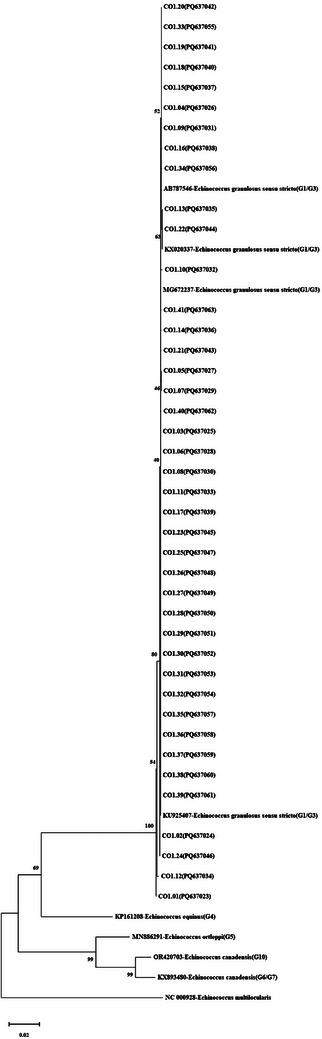
Phylogram of *Echinococcus granulosus* s.l. isolates using the maximum likelihood method. Phylogenetic tree was constructed using mt‐*CO1* gene (1603 bp) sequences and the reference sequences *E. granulosus* s.s. (G1/G3) (AB787546, KX020337, MG672237, KU925407), *E. equinus* (G4) (KP161208), *E. ortleppi* (G5) (MN886291), *E. canadensis* (G10) (OR420703), and *E. canadensis* (G6/G7) (KX893480), and the outgroup sequence *E. multilocularis* (NC000928). It was created based on the TN93+G model with the Maximum Likelihood method in MEGA‐X, and the reliability of the tree was ensured by 1000 bootstrap tests.

**FIGURE 2:**
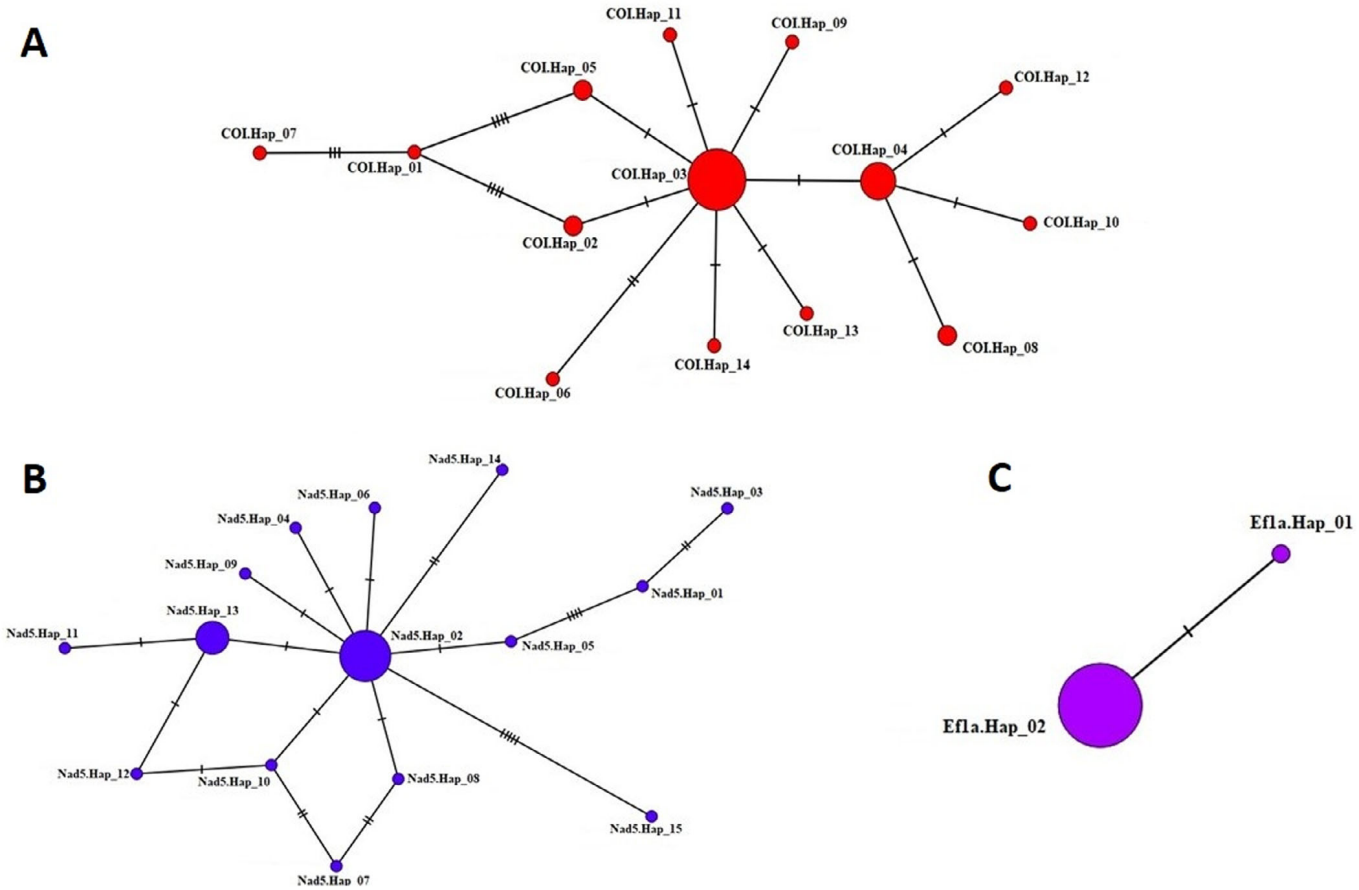
Haplotype network for mt‐*CO1* (A), mt‐*Nad5* (B), and *ef1α* (C) genes of *Echinococcus granulosus* s.s. (G1/G3) sheep isolates. The size of the circles is proportional to the frequency of each haplotype. The number of mutations separating haplotypes is indicated by dashes. Hap: haplotype.

In 41 hydatid cyst samples, the mt‐*CO1* gene region was amplified using PCR to definitively determine the species. PCR results showed a band profile of 1810 bp in all 41 isolates. Pairwise DNA sequence analysis was conducted on these samples. Forward and reverse sequences for each isolate were analyzed using FinchTV 1.4.0 (Geospiza Inc., Seattle, Washington, USA) (http://www.geospiza.com). The sequence ends were trimmed by comparing them with published sequences through the ‘BLAST’ (http://www.ncbi.nlm.nih.gov/BLAST/) search. The trimmed sequences had a final size of 1603 bp for the 41 sequences (CO1.01–CO1.41). BLAST analysis confirmed that all isolates were *E. granulosus* s.s. (G1/G3). These sequences were deposited in the NCBI database, and the accession numbers are shown in Table S1. The most suitable phylogenetic tree model for the sequences was determined to be TN93+G (Tamura‐Nei+Gamma distribution (+G)) in the MEGA X program, and the phylogenetic tree was constructed using the maximum likelihood statistical method with a bootstrap test (1000 repetitions). The phylogenetic tree of the 1603 bp fragment of the mt‐*CO1* gene sequences (*n* = 41), along with the reference and outgroup sequences, is shown in Figure 1. The haplotype network revealed two main haplotypes (CO1.Hap_03 and CO1.Hap_04) covering 63.41% (26/41) of the isolates and 14 haplotypes separated from these main haplotypes by 1–9 mutation steps (Figure 2A). Seventeen polymorphic sites were identified for mt‐*CO1* sequences; 35.29% (6/17) were parsimony informative, indicating high haplotype and low nucleotide diversity for this gene region. Tajima's *D*, which indicates population expansion and/or purifying selection, was negative. Significantly negative Fu's *F*s values indicated an excess number of alleles, which would be expected in the case of recent population expansion. Negative and significant FLD and FLF values also supported the identification of rare haplotypes due to population expansion (Table S2). The results were further supported by the fact that 64.28% (9/14) of the haplotype groups were single haplotypes.

### 3.2 mt‐*Nad5* Sequences and Haplotype Findings

**FIGURE 3:**
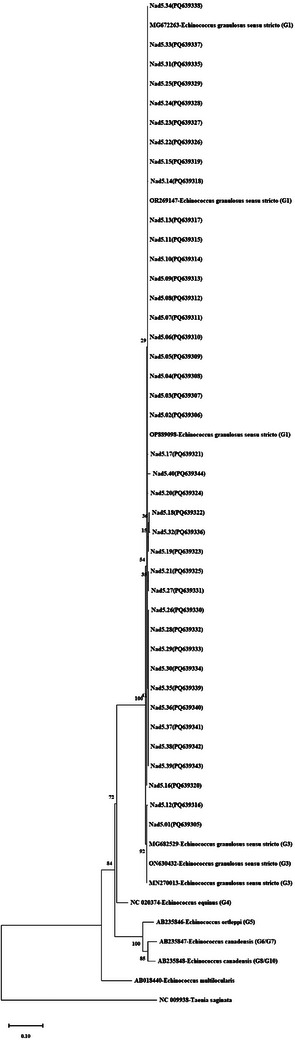
Phylogenetic tree of *Echinococcus granulosus* isolates using the maximum likelihood method. Phylogenetic tree was constructed using mt‐*Nad5* gene (759 bp) sequences and the reference sequences *E. granulosus* s.s. (G1/G3) (MG672263, OR269147, OP889098, MG682529, ON630432, MN270013), *E. equinus* (G4) (NC020374), *E. ortleppi* (G5) (AB235846), *E. canadensis* (G8/G10) (AB235848), and *E. canadensis* (G6/G7) (AB235847) with outgroup sequences of *E. multilocularis* (AB018440) and *T. saginata* (NC009938). It was created based on the HKY+I model with the Maximum Likelihood method in MEGA‐X, and the reliability of the tree was ensured by 1000 bootstrap tests.

The mt‐*Nad5* (759 bp) gene region was amplified by PCR to differentiate between G1 and G3 genotypes in samples identified as *E. granulosus* s.s. (G1/G3). PCR results showed a 759‐bp band profile in 40 isolates, while one isolate did not yield a band sufficient for sequence analysis despite repeated attempts. One‐way DNA sequence analysis was performed on these samples, and after trimming the sequence ends, the final size of the sequences was 625 bp for 40 sequences (Nad5.01–Nad5.40). BLAST analysis identified 38 isolates as G1 and 2 isolates as G3. Alignment with the reference sequence of the G1 genotype (AB786664) revealed G3‐specific point mutations in isolates Nad5.01 and Nad5.12 as described by Kinkar et al. 2018a). All sequences were submitted to NCBI, and accession numbers were obtained, as shown in Table S3. The phylogenetic tree model (HKY+I) was determined to be the most suitable phylogenetic tree model for the sequences in the MEGA X program, and the phylogenetic tree was constructed with the bootstrap test (1000 replicates) using the maximum likelihood statistical method (Figure 3). The resulting haplotype network identified 15 haplotypes, organized with two main haplotypes (Nad5.Hap_02 and Nad5.Hap_13), separated from other haplotypes by 1–8 mutation steps and covering 67.5% (27/40) of the total isolates (Figure 2B). For mt‐*Nad5* sequences, 20 polymorphic sites were identified, 45% (9/20) of which were parsimony informative, indicating high haplotype and almost low nucleotide diversity for this gene region. Tajima's *D* was negative and significant, indicating population expansion and/or slowed selection. The observed significantly negative Fu's *F*s values supported a recent population expansion. Negative values in FLD and FLF statistics also supported population expansion (Table S4). The results were further supported by the fact that 86.66% (13/15) of the haplotype groups were single haplotypes.

### 3.3 *Ef1α* Sequences and Haplotype Findings

**FIGURE 4:**
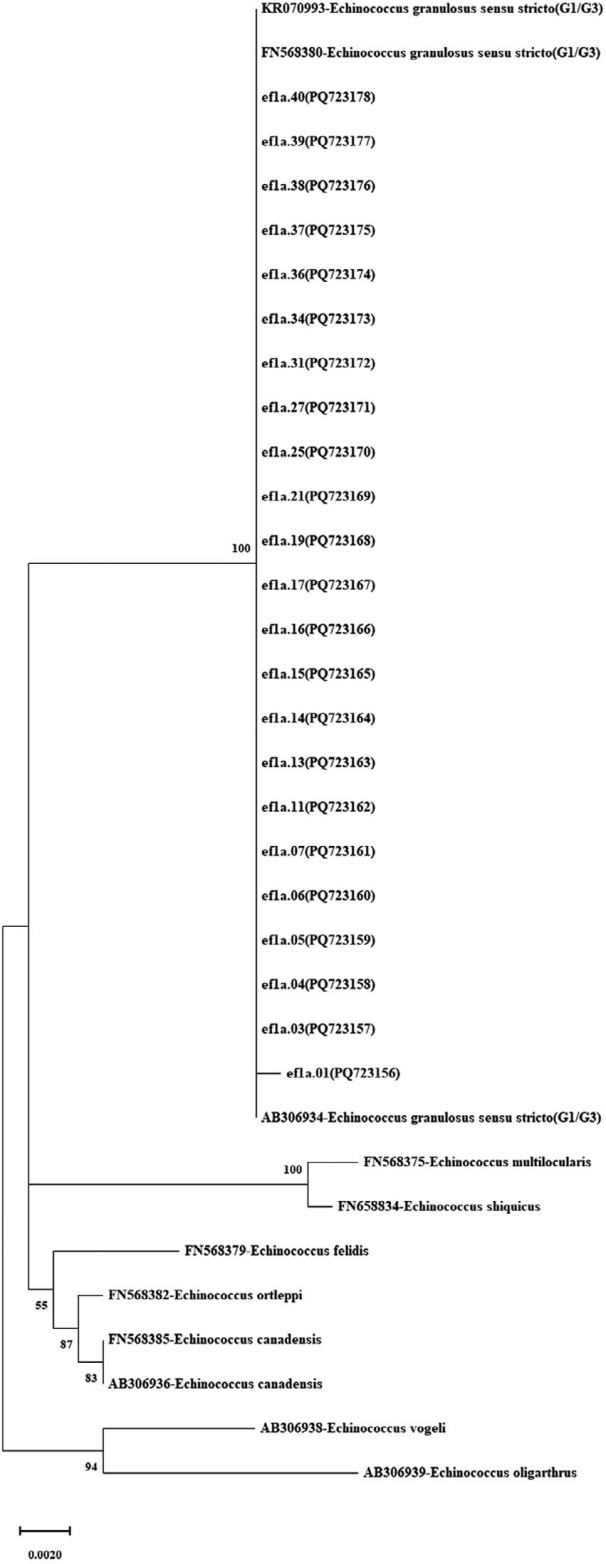
Phylogenetic tree view constructed using 985‐bp nuclear *ef1α* gene sequences of *Echinococcus granulosus* isolates, reference sequences *E. granulosus* s.s. (G1/G3) (KR070993, FN568380, AB306934), *E. ortleppi* (G5) (FN568382), *E. canadensis* (FN568385), and *E. canadensis* (AB306936). Outgroup sequences: *E. multilocularis* (FN568375), *E. shiquicus* (FN658834), *E. felidis* (FN568379), *E. vogeli* (AB306938), and *E. oligarthrus* (AB306939). It was created based on the JC model with the maximum likelihood method in MEGA‐X, and the reliability of the tree was ensured by 1000 bootstrap tests.

PCR resulted in a band profile of 1343 bp in 23 of the 41 isolates, and unidirectional DNA sequence analysis was conducted. Sequence ends were trimmed by comparing them with published sequences. The final size of the trimmed sequences was 985 bp for 23 sequences (ef1a.01–ef1a.40). These 23 *ef1α* sequences were deposited in the NCBI database, and the accession numbers are shown in Table S5. The most appropriate phylogenetic tree model for the sequences was determined to be the K2 model (Kimura 2 parameter), and the phylogenetic tree was constructed using the maximum likelihood statistical method with a bootstrap test (1000 replicates). The phylogenetic tree for the 985 bp fragment of the *ef1α* gene (*n* = 23), along with reference and outgroup sequences, is shown in Figure 4. Haplotype analysis of the 23 *ef1α* sequences among the sheep isolates obtained in this study was performed. The haplotype network analysis revealed that the main haplotype (ef1a.Hap_02) accounted for 95.65% (22/23) of the isolates, forming a network. Two haplotypes were detected in total, with one single haplotype separated from the main haplotype by one mutation step (Figure 2C). No parsimony informative sites were identified for the *ef1α* sequences, but one polymorphic site (849th nucleotide) was detected, and very low haplotype and nucleotide diversity were observed. Negative but insignificant Tajima's *D* and Fu's *F*s values indicated the presence of extremely rare mutations. This was also supported by FLD and FLF statistics showing negative values (Table S6).

## 4 Discussion

In this study, a larger gene region (1603 bp) was selected for DNA sequence analysis of sheep isolates, unlike the partial mt‐*CO1* gene regions (446 and 857 bp) commonly used in other studies, to monitor intraspecies microvariations and haplotypes and to make definitive species determination. The mt‐*Nad5* gene region was employed to strengthen the genotyping results from the mt‐*CO1* region and to reveal the genotype distinction within the *E. granulosus* s.s. (G1/G3) species. Additionally, the effects of variations in the nuclear *ef1α* gene region on *Echinococcus* phylogeny and haplotype diversity were evaluated. Various studies using different DNA markers have previously shed light on genetic diversity and intraspecific variation in the genus *Echinococcus* (Bowles et al. 1992; Bowles et al. 1993; Nakao et al. 2010; Boufana et al. 2014).

In the present study, the results obtained using mt‐DNA support previous observations (Simsek et al. 2011; Kesik et al. 2019; Celik et al. 2024; Selcuk et al. 2024) that the G1 genotype of *E. granulosus* s.s. is the predominant species. In an effort to assess the genetic diversity and haplotype profiles of hydatid cysts from cattle lungs in three provinces in eastern Türkiye, PCR amplification of a partial mt‐*CO1* gene was performed on 60 hydatid cyst isolates, identifying all as *E. granulosus* s.s. (G1/G3). Forty‐nine point mutations were detected, and 33 haplotypes were identified in 60 samples (Kesik et al. 2022). In this study, 17 polymorphic sites (Table S7) were identified as a result of mt‐*CO1* gene region (1603 bp) sequence analysis, revealing 14 haplotypes.

Molecular characterization studies using partial mt‐*CO1* gene regions (such as 446 bp, 503 bp, and 758 bp) have reported lower haplotype numbers and diversity compared to larger gene regions (Mehmood et al. 2020). However, in this study, even though the 1603‐bp mt‐*CO1* gene region was used, a lower haplotype number (*n* = 14) was identified. This was interpreted as being caused by the hydatid cyst samples primarily coming from the same herd. The fact that the samples from Elazig province were mostly from a single herd may have contributed to this observation. Additionally, examining the diversity and neutrality indices obtained from the mt‐*CO1* gene (1603 bp) nucleotide data of *E. granulosus* s.s. (G1/G3) revealed significantly negative Tajima's *D* values (−1.94023), indicating variable nucleotides and population expansion (*p* < 0.05) (Boufana et al. 2015; Cengiz et al. 2020; Kesik et al. 2021; Kesik et al. 2022). In a dataset consisting of 35 hydatid cysts belonging to the CO1 gene region, which was obtained from Iran, haplotype diversity (Hd) changes as high, while nucleotide diversity changes as low. When Tajima's *D* and Fu's *F*s tests were performed, the neutrality indices that were obtained were negative (Yanagida et al. 2012). The results of a study that was carried out in Iraq revealed that there was a significant haplotype diversity but a low nucleotide diversity among the 36 isolates. Tajima's *D* value, which was positive but not significant, was regarded as indicating that population growth was occurring (Al‐Hizab et al. 2021). This result was supported by Fu's *F*s value, which was also negative and significant, suggesting the emergence of extra alleles as expected after recent population expansion or genetic hitchhiking. These negative and significant values of Tajima's *D* and Fu's *F*s neutrality indices suggest possible recent bottleneck events.

In another study aiming to evaluate and compare the genotypic diversity of *E. granulosus* in livestock in Türkiye and Iran, DNA isolation and partial sequence analysis of mt‐*CO1* and *nad1* gene regions were performed from isolates obtained from Iran (60 samples including 30 sheep and 30 cattle) and Türkiye (30 samples including 15 sheep and 15 cattle). Five different haplotypes were found in the sheep and cattle isolates from both countries, with all isolates clustered in one group (Barazesh et al. 2019). In the present study, a sequence from each haplotype cluster was compared with other sequences in NCBI. One of the two main haplotype clusters with the highest frequency, CO1.Hap_03, was 100% similar to sheep isolates from France, Spain, Argentina, Greece, and Türkiye (accession numbers MG672124, MG672282, KX039943, and MG672149). The other main haplotype CO1.Hap_04 was 100% similar to sheep isolates from Algeria, Italy, Armenia, Türkiye, and Argentina. Based on these data, it can be concluded that the sheep isolates in the present study share a common genetic ancestor with *E. granulosus* populations from various geographical regions.

With the development of molecular methods, nominal species have been re‐evaluated, and latent species that are morphologically identical but genetically distinct have been revealed. Over time, due to speciation diversity in parasitic helminths, latent diversities have emerged in large parasite and host communities (Cháves‐González et al. 2022). When partial cox and nad sequences are used, mt‐DNA alone is not sufficient to identify latent species due to the inability to discriminate intraspecific variability resulting from monophyly (Blouin 2002). Therefore, additional markers and larger DNA fragments have been proposed to provide higher resolution of species taxonomic position (Blouin 2002). For cestodes, mitochondrial markers such as cox and nad have been used to study latent diversity in the Taeniidae family (Lavikainen et al. 2010; Jia et al. 2012). Since discrepancies can be observed between mitogenome and nuclear gene phylogenies, it is recommended to apply several markers together for a more robust interpretation of phylogenetic relationships. Therefore, both mitochondrial and nuclear markers were used together for phylogenetic analyses in the present study. Reports indicate that molecular analysis of the mt‐*CO1* gene region should be supplemented by other gene regions (Laurimäe et al. 2018). In particular, the mt‐*Nad5* gene region effectively differentiates G1 and G3 genotypes due to specific nucleotide changes (Kinkar et al. 2018). In this study, the mt‐*Nad5* gene region was used to enhance genotyping results from the mt‐*CO1* region and to detail the genotype distinction within *E. granulosus* s.s. (G1/G3) species. The specific nucleotide differences reported by Kinkar et al. (2018a) were also identified in this study (Table S8).

DNA sequence analysis of the mt‐*N*ad5 (759 bp) gene region from a total of 40 sheep isolates identified 38 isolates as G1 and two isolates as G3. Additionally, 15 different haplotypes were detected among these isolates, with 13 belonging to the G1 genotype and two to the G3 genotype. This study revealed high intraspecific haplotype diversity and high nucleotide diversity, with 20 different nucleotide variation positions identified among the haplotypes. The higher number of polymorphic areas and haplotypes in G1 compared to G3 is consistent with other studies (Shahabi et al. 2021; Samari et al. 2022; Celik et al. 2024). Previous studies in Türkiye have also shown specific nucleotide differences between genotypes through sequence analysis of the mt‐*Nad5* gene regions, with G1 being identified as the dominant genotype (Cengiz et al. 2020; Shahabi et al. 2021). Following the completion of a study in Iraq, partial Nad5 gene sections were amplified from a total of 12 different isolates; however, only one of the isolates was found to have the G3 genotype (Farhood et al. 2024).

A global study analyzing the genetic diversity of *E. granulosus* s.s. (G1/G3) isolates through mitochondrial genome sequences from 222 samples across 22 countries revealed 212 G1 and 10 G3 isolates, highlighting significant genetic diversity within the G1 genotype (Kinkar et al. 2018b). The current distribution of the G1 genotype has been influenced by extensive animal movements, as shown by comprehensive phylogenetic geographic model analyses (Kinkar et al. 2018b). The study successfully distinguished specific nucleotide differences and intraspecific genotypes using the mt‐*nad5* gene sequence, noting high haplotype and nucleotide diversity within the genotypes. Therefore, the regular monitoring of genetic variations in *E. granulosus* s.s. (G1/G3) is important in countries such as Türkiye, where CE is common in sheep, to track phylogeographic data (Celik et al. 2024).

Interconnected nature of genes within the mitochondrial genome means that different mt‐DNA genes typically do not satisfy the statistical independence necessary for accurately representing the evolutionary history of genomes as a whole, rather than just single loci (Ballard et al. 2005). This study also utilized the nuclear *ef1α* gene for genetic diversity analysis. DNA sequence analysis of this gene showed low nucleotide diversity in the analyzed samples, with one point mutation (at nucleotide 849) detected, resulting in a total of two haplotypes (Table S9). Although nuclear genes exhibit several positive characteristics, it is important not to overlook the potential for paralogous copies of these genes leading to erroneous phylogenies (Saarma et al. 2009). Until recently, limited studies analyzing multiple nuclear loci to infer the phylogeny of *E. granulosus* s.l. have yielded conflicting results (Saarma et al. 2009; Knapp et al. 2011; Laurimäe et al. 2018). For instance, Saarma et al. (2009) used sequences from five different nuclear gene markers for phylogenetic analysis of *Echinococcus* species, revealing a significantly different phylogeny but aligning with previous species classifications. Knapp et al. (2011) suggested that the *E. granulosus* s.l. complex could be paraphyletic based on varied nuclear loci, which corresponded with mt‐DNA data. Laurimäe (2018) aimed to present definitive evidence for treating G1/G3 genotypes as a single species by sequencing three nuclear gene regions (ef1, tgf, and cal). Furthermore, nearly complete mitogenomes were sequenced for the same sample set to accurately assign samples to the correct genotypes, since short sequences of mt‐DNA sometimes fail to sufficiently distinguish between G1/G3 genotypes. Their mt‐DNA phylogenetic network analysis revealed that G2 samples clustered within the G3 haplogroup, suggesting that G2 might not be a valid genotype within *E. granulosus* s.s. (G1/G3). G1 and G3 genotypes, differentiated by 37 mutations, formed two distinct clusters. However, the nuclear data did not distinguish between the G1 and G3 genotype clusters, leading to the conclusion that G1 and G3 can be considered a single species (*E. granulosus* s.s.) and are separate genotypes only in the context of mitochondrial data (Laurimäe 2018).

In this study, the G1 and G3 genotypes within *E. granulosus* s.s., distinguished by the mt‐*Nad5* gene region of sheep hydatid cyst isolates, also formed separate haplogroups as a result of haplotype analysis of the *ef1α* nuclear gene. The ef1α.Hap_01 haplotype was exclusive to the G3 genotype sequence, while the other G1 genotypes were within the main haplotype ef1α.Hap_02. This indicates that the nuclear *ef1α* gene region is similar to the mt‐*Nad5* gene region in distinguishing (G1/G3) intraspecific genotypes, supporting the DNA sequence analysis results. The *ef1α* nuclear gene sequence analysis revealed a limited number of nucleotide variations among sheep hydatid cyst isolates, with the mutation at nucleotide 849 (A‐G) indicating the distinction of the G3 isolate (ef1α.01) from the ef1α.Hap_02 main haplotype formed. It is important to note that the sample size for the *ef1α* gene region (*n* = 23) was smaller compared to the mt‐DNA markers, such as mt‐*CO1* (*n* = 41) and mt‐*Nad5* (*n* = 40). This smaller sample size may limit the resolution of genetic diversity findings for *ef1α*, particularly in identifying rare mutations and distinguishing finer population structure. While the *ef1α* sequences still provided valuable insights into haplotype diversity, future studies with a larger sample size could enhance our understanding of the genetic variation and evolutionary dynamics of *Echinococcus granulosus* in this region.

## 5 Conclusion

As a result, when evaluating the phylogenies formed by sequences of different gene regions of *E. granulosus* s.s. (G1/G3) isolates, it was observed that the phylogenetic tree created with the mt‐*CO1* and *Nad5* gene dataset placed the G1 and G3 isolates in separate clades with the reference sequences. This finding is consistent with previous studies on the phylogeny of *E. granulosus* s.s. (G1/G3) (Nakao et al. 2013; Romig et al. 2015; Kinkar et al. 2018a). However, in the phylogenetic tree created with the nuclear gene region *ef1α* dataset, unlike the mitochondrial genes, no complete monophyly was observed. The isolate belonging to the G3 genotype (ef1a.01) formed a sister clade with the G1 genotypes and the reference sequence. The limited number of sequences from the G3 genotype used in this study poses some interpretative limitations. Therefore, the nuclear gene‐based phylogeny results align with previous phylogeny studies (Saarma et al. 2009; Laurimäe 2018) showing that although the maternal lineages of the isolates in this study were quite distinct, their nuclear structures were highly similar. Future studies could determine a higher rate of nuclear gene polymorphism through molecular characterization of larger sample sizes and wider gene regions, aiding in the understanding of the effect of *E. granulosus* s.l. on intraspecific variations.

## Author Contributions

Seyma Gunyakti Kilinc: Data curation, formal analysis, investigating, writing—review and editing. Harun Kaya Kesik: Funding acquisition, methodology, project administration, writing—original draft. Figen Celik: Resources, validation, visualization, writing—review and editing. Sami Simsek: Methodology, supervision, investigation, writing—review and editing.

## Ethics Statement

According to the decision of Bingöl University Animal Experiments Local Ethics Committee dated 11.08.2023 and numbered E‐85680299‐020‐117583, ‘Procedures performed with dead animals or tissues, slaughterhouse materials, waste foetuses are not subject to ethics committee permission’, it was decided that ethics committee permission was not required.

## Conflicts of Interest

The authors declare no conflicts of interest.

## Supporting information



Supporting information

## Data Availability

All data generated or analyzed during this study are included in this article. Data is available within the article.
