# Supplementary material for: Comparative Analysis of Nuclear (Ef1α) and Mitochondrial (mt‐CO1 and mt‐Nad5) Markers for Molecular Characterization of Sheep Isolates of Echinococcus granulosus sensu lato
Source: Vet Med Sci. 2025 Mar 20;11(2):e70313. doi: 10.1002/vms3.70313 (PMC11923390; doi:10.1002/vms3.70313)
Supplement: Supplementary file 1 — Supporting information [file VMS3-11-e70313-s001.docx]

**Supplementary Tables**

**Table 1**. Accession numbers of mt-CO1 gene region sequences.

| **Isolate number** | **Isolate name** | **Fragment length** | **Acc. No.** |
| --- | --- | --- | --- |
| 1 | CO1.01 | 1603 bp | PQ637023 |
| 2 | CO1.02 | 1603 bp | PQ637024 |
| 3 | CO1.03 | 1603 bp | PQ637025 |
| 4 | CO1.04 | 1603 bp | PQ637026 |
| 5 | CO1.05 | 1603 bp | PQ637027 |
| 6 | CO1.06 | 1603 bp | PQ637028 |
| 7 | CO1.07 | 1603 bp | PQ637029 |
| 8 | CO1.08 | 1603 bp | PQ637030 |
| 9 | CO1.09 | 1603 bp | PQ637031 |
| 10 | CO1.10 | 1603 bp | PQ637032 |
| 11 | CO1.11 | 1603 bp | PQ637033 |
| 12 | CO1.12 | 1603 bp | PQ637034 |
| 13 | CO1.13 | 1603 bp | PQ637035 |
| 14 | CO1.14 | 1603 bp | PQ637036 |
| 15 | CO1.15 | 1603 bp | PQ637037 |
| 16 | CO1.16 | 1603 bp | PQ637038 |
| 17 | CO1.17 | 1603 bp | PQ637039 |
| 18 | CO1.18 | 1603 bp | PQ637040 |
| 19 | CO1.19 | 1603 bp | PQ637041 |
| 20 | CO1.20 | 1603 bp | PQ637042 |
| 21 | CO1.21 | 1603 bp | PQ637043 |
| 22 | CO1.22 | 1603 bp | PQ637044 |
| 23 | CO1.23 | 1603 bp | PQ637045 |
| 24 | CO1.24 | 1603 bp | PQ637046 |
| 25 | CO1.25 | 1603 bp | PQ637047 |
| 26 | CO1.26 | 1603 bp | PQ637048 |
| 27 | CO1.27 | 1603 bp | PQ637049 |
| 28 | CO1.28 | 1603 bp | PQ637050 |
| 29 | CO1.29 | 1603 bp | PQ637051 |
| 30 | CO1.30 | 1603 bp | PQ637052 |
| 31 | CO1.31 | 1603 bp | PQ637053 |
| 32 | CO1.32 | 1603 bp | PQ637054 |
| 33 | CO1.33 | 1603 bp | PQ637055 |
| 34 | CO1.34 | 1603 bp | PQ637056 |
| 35 | CO1.35 | 1603 bp | PQ637057 |
| 36 | CO1.36 | 1603 bp | PQ637058 |
| 37 | CO1.37 | 1603 bp | PQ637059 |
| 38 | CO1.38 | 1603 bp | PQ637060 |
| 39 | CO1.39 | 1603 bp | PQ637061 |
| 40 | CO1.40 | 1603 bp | PQ637062 |
| 41 | CO1.41 | 1603 bp | PQ637063 |
|  |  |  |  |

**Table 2**. Diversity and neutrality indices obtained using nucleotide data of the mt-CO1 gene (1603 bp) of Echinococcus granulosus s.s. (G1/G3).

| n | H | hd±SD | πd±SD | Tajima’s D | p value | Fu’s Fs | p value | FLD | p value | FLF | p value |
| --- | --- | --- | --- | --- | --- | --- | --- | --- | --- | --- | --- |
| 41 | 14 | 0,762±0,063 | 0,00099±0,00021 | -1,94023 | *P<0.05 | -8,607 | 0,000 | -2,73510 | *P<0.05 | -2,91840 | *P<0.05 |

**Table 3**. Accession numbers of nad5 gene region sequences.

| **Isolate number** | **Isolate name** | **Fragment length** | **Acc. No.** |
| --- | --- | --- | --- |
| 1 | Nad5.01 | 625 bp | PQ639305 |
| 2 | Nad5.02 | 625 bp | PQ639306 |
| 3 | Nad5.03 | 625 bp | PQ639307 |
| 4 | Nad5.04 | 625 bp | PQ639308 |
| 5 | Nad5.05 | 625 bp | PQ639309 |
| 6 | Nad5.06 | 625 bp | PQ639310 |
| 7 | Nad5.07 | 625 bp | PQ639311 |
| 8 | Nad5.08 | 625 bp | PQ639312 |
| 9 | Nad5.09 | 625 bp | PQ639313 |
| 10 | Nad5.10 | 625 bp | PQ639314 |
| 11 | Nad5.11 | 625 bp | PQ639315 |
| 12 | Nad5.12 | 625 bp | PQ639316 |
| 13 | Nad5.13 | 625 bp | PQ639317 |
| 14 | Nad5.14 | 625 bp | PQ639318 |
| 15 | Nad5.15 | 625 bp | PQ639319 |
| 16 | Nad5.16 | 625 bp | PQ639320 |
| 17 | Nad5.17 | 625 bp | PQ639321 |
| 18 | Nad5.18 | 625 bp | PQ639322 |
| 19 | Nad5.19 | 625 bp | PQ639323 |
| 20 | Nad5.20 | 625 bp | PQ639324 |
| 21 | Nad5.21 | 625 bp | PQ639325 |
| 22 | Nad5.22 | 625 bp | PQ639326 |
| 23 | Nad5.23 | 625 bp | PQ639327 |
| 24 | Nad5.24 | 625 bp | PQ639328 |
| 25 | Nad5.25 | 625 bp | PQ639329 |
| 26 | Nad5.26 | 625 bp | PQ639330 |
| 27 | Nad5.27 | 625 bp | PQ639331 |
| 28 | Nad5.28 | 625 bp | PQ639332 |
| 29 | Nad5.29 | 625 bp | PQ639333 |
| 30 | Nad5.30 | 625 bp | PQ639334 |
| 31 | Nad5.31 | 625 bp | PQ639335 |
| 32 | Nad5.32 | 625 bp | PQ639336 |
| 33 | Nad5.33 | 625 bp | PQ639337 |
| 34 | Nad5.34 | 625 bp | PQ639338 |
| 35 | Nad5.35 | 625 bp | PQ639339 |
| 36 | Nad5.36 | 625 bp | PQ639340 |
| 37 | Nad5.37 | 625 bp | PQ639341 |
| 38 | Nad5.38 | 625 bp | PQ639342 |
| 39 | Nad5.39 | 625 bp | PQ639343 |
| 40 | Nad5.40 | 625 bp | PQ639344 |
|  |  |  |  |

**Table 4**. Diversity and neutrality indices obtained using nucleotide data of the mt-nad5 gene (625 bp) of Echinococcus granulosus s.s. (G1/G3).

| n | H | hd±SD | πd±SD | Tajima’s D | p value | Fu’s Fs | p value | FLD | p value | FLF | p value |
| --- | --- | --- | --- | --- | --- | --- | --- | --- | --- | --- | --- |
| 40 | 15 | 0,745±0,065 | 0,00295±0,00070 | -2,00882 | *,P<0.05 | -8,957 | 0,000 | -2,12953 | 0.10>P>0.05 | -2,46970 | 0.10>P>0.05 |

**Table 5**. Accession numbers of ef1α gene region sequences

| **Isolate number** | **Isolate name** | **Fragment length** | **Acc. No.** |
| --- | --- | --- | --- |
| 1 | ef1a.01 | 985 bp | PQ723156 |
| 2 | ef1a.03 | 985 bp | PQ723157 |
| 3 | ef1a.04 | 985 bp | PQ723158 |
| 4 | ef1a.05 | 985 bp | PQ723159 |
| 5 | ef1a.06 | 985 bp | PQ723160 |
| 6 | ef1a.07 | 985 bp | PQ723161 |
| 7 | ef1a.11 | 985 bp | PQ723162 |
| 8 | ef1a.13 | 985 bp | PQ723163 |
| 9 | ef1a.14 | 985 bp | PQ723164 |
| 10 | ef1a.15 | 985 bp | PQ723165 |
| 11 | ef1a.16 | 985 bp | PQ723166 |
| 12 | ef1a.17 | 985 bp | PQ723167 |
| 13 | ef1a.19 | 985 bp | PQ723168 |
| 14 | ef1a.21 | 985 bp | PQ723169 |
| 15 | ef1a.25 | 985 bp | PQ723170 |
| 16 | ef1a.27 | 985 bp | PQ723171 |
| 17 | ef1a.31 | 985 bp | PQ723172 |
| 18 | ef1a.34 | 985 bp | PQ723173 |
| 19 | ef1a.36 | 985 bp | PQ723174 |
| 20 | ef1a.37 | 985 bp | PQ723175 |
| 21 | ef1a.38 | 985 bp | PQ723176 |
| 22 | ef1a.39 | 985 bp | PQ723177 |
| 23 | ef1a.40 | 985 bp | PQ723178 |

| n | H | hd±SD | πd±SD | Tajima’s D | p value | Fu’s Fs | p value | FLD | p value | FLF | p value |
| --- | --- | --- | --- | --- | --- | --- | --- | --- | --- | --- | --- |
| 23 | 2 | 0,087±0,078 | 0,00009±0,00008 | -1,16097 | P> 0.10 | -0,993 | 0,234 | -1,59071 | P > 0.10 | -1,69158 | P > 0.10 |

**Table 6**. Diversity and neutrality indices obtained using nucleotide data of the Elongation Factor 1 Alpha (ef1α) gene (985 bp) of *Echinococcus granulosus*

**Table 7:** Nucleotide variation positions of the mt-CO1 gene (1603 bp) among the 14 haplotypes

| **Nucleotide**  **Positions** | **42** | **189** | **321** | **504** | **505** | **590** | **717** | **800** | **810** | **1001** | **1245** | **1458** | **1485** | **1518** | **1536** | **1547** | **1592** |
| --- | --- | --- | --- | --- | --- | --- | --- | --- | --- | --- | --- | --- | --- | --- | --- | --- | --- |
| NC_044548 (reference) | T | C | T | T | C | T | C | C | C | T | T | G | T | T | C | C | C |
| CO1.Hap_01 |  |  |  |  |  |  | T |  | T | C |  |  |  |  | T |  | T |
| CO1.Hap_02 |  |  |  |  |  |  |  |  |  |  |  |  |  |  | T |  |  |
| CO1.Hap_03 |  |  |  |  |  |  |  |  |  |  |  |  |  |  |  |  |  |
| CO1.Hap_04 |  |  |  |  |  |  |  |  |  |  |  |  |  |  |  |  |  |
| CO1.Hap_05 |  |  |  |  |  |  |  |  | T |  |  |  |  |  |  |  |  |
| CO1.Hap_06 |  |  | C |  |  |  |  |  |  |  |  |  | C |  |  |  |  |
| CO1.Hap_07 |  |  |  |  |  |  |  | T | T | C |  |  |  | C | T | T | T |
| CO1.Hap_08 |  | T |  |  |  |  |  | T |  |  |  |  |  |  |  |  |  |
| CO1.Hap_09 |  |  |  | C |  |  |  |  |  |  |  |  |  |  |  |  |  |
| CO1.Hap_10 |  |  |  |  |  |  |  | T |  |  |  | A |  |  |  |  |  |
| CO1.Hap_11 | C |  |  |  |  |  |  |  |  |  |  |  |  |  |  |  |  |
| CO1.Hap_12 |  |  |  |  |  |  |  | T |  |  | C |  |  |  |  |  |  |
| CO1.Hap_13 |  |  |  |  | T |  |  |  |  |  |  |  |  |  |  |  |  |
| CO1.Hap_14 |  |  |  |  |  | G |  |  |  |  |  |  |  |  |  |  |  |

**Table 8:** Nucleotide variation positions of the mt-nad5 gene (625 bp) among the 15 haplotypes

| **Nucleotide**  **Positions** | **17** | **20** | **40** | **182** | **210** | **271** | **338** | **352** | **359** | **365** | **369** | **421** | **445** | **448** | **487** | **533** | **560** | **577** | **607** | **616** |
| --- | --- | --- | --- | --- | --- | --- | --- | --- | --- | --- | --- | --- | --- | --- | --- | --- | --- | --- | --- | --- |
| NC_044548 (reference) | A | C | T | C | T | C | T | A | G | T | T | G | G | G | T | T | G | T | A | G |
| Nad5.Hap_01 | G |  |  |  |  | T |  |  | A |  |  |  |  |  |  |  |  |  | G | A |
| Nad5.Hap_02 |  |  |  |  |  |  |  |  |  |  |  |  |  |  |  |  |  |  |  |  |
| Nad5.Hap_03 | G |  |  |  |  | T |  |  | A |  |  |  |  | A | G |  |  |  | G | A |
| Nad5.Hap_04 |  |  |  |  |  |  |  |  |  |  |  |  |  |  |  | C |  |  |  |  |
| Nad5.Hap_05 |  |  |  |  |  | T |  |  |  |  |  |  |  |  |  |  |  |  |  |  |
| Nad5.Hap_06 |  |  |  | T |  |  |  |  |  |  |  |  |  |  |  |  |  |  |  |  |
| Nad5.Hap_07 |  |  |  |  |  |  |  |  |  |  |  |  | A | A |  |  | A |  |  |  |
| Nad5.Hap_08 |  |  |  |  |  |  |  |  |  |  |  |  |  |  |  |  |  |  |  |  |
| Nad5.Hap_09 |  |  |  |  |  |  |  |  |  |  |  | A |  |  |  |  |  |  |  |  |
| Nad5.Hap_10 |  |  |  |  |  |  |  |  |  |  |  |  |  | A |  |  |  |  |  |  |
| Nad5.Hap_11 |  |  |  |  |  |  |  | G |  |  |  |  |  |  |  |  |  | G |  |  |
| Nad5.Hap_12 |  |  |  |  |  |  |  | G |  |  |  |  |  | A |  |  |  |  |  |  |
| Nad5.Hap_13 |  |  |  |  |  |  |  | G |  |  |  |  |  |  |  |  |  |  |  |  |
| Nad5.Hap_14 |  | T |  |  |  |  |  |  |  |  |  |  | A |  |  |  |  |  |  |  |
| Nad5.Hap_15 |  |  | C |  | C |  | C |  |  | C | A |  |  |  |  |  |  |  |  |  |

**Table 9:** Nucleotide variation positions of the Elongation Factor 1 Alpha (ef1α) gene (985 bp) between the 2 haplotypes

| **Nucleotide**  **Positions** | **849** |
| --- | --- |
| > KR070993  (reference) | G |
| ef1α.Hap_01 | A |
| ef1α.Hap_02 | G |
